# Absolute pitch in involuntary musical imagery

**DOI:** 10.3758/s13414-024-02936-0

**Published:** 2024-08-12

**Authors:** Matthew G. Evans, Pablo Gaeta, Nicolas Davidenko

**Affiliations:** 1 Department of Psychology, University of California Santa Cruz, Santa Cruz, CA USA; 2 Department of Computer Science and Engineering, UC Santa Cruz, Santa Cruz, CA USA

**Keywords:** Absolute pitch, Earworms, Involuntary musical imagery, INMI, Music cognition, Auditory imagery

## Abstract

Memory for isolated absolute pitches is extremely rare in Western, English-speaking populations. However, past research has found that people can voluntarily reproduce well-known songs in the original key much more often than chance. It is unknown whether this requires deliberate effort or if it manifests in involuntary musical imagery (*INMI*, or earworms). Participants (*N* = 30, convenience sample) were surveyed at random times over a week and asked to produce a sung recording of any music they were experiencing in their heads. We measured the “pitch error” of each recording to the nearest semitone by comparing participants’ recordings to the original song. We found that 44.7% of recordings had a pitch error of 0 semitones, and 68.9% of recordings were within ± 1 semitone of the original song. Our results provide novel evidence that a large proportion of the population has access to absolute pitch, as revealed in their INMI.

**Supplementary Information:**

The online version contains supplementary material available at 10.3758/s13414-024-02936-0.

If you’re walking down the street and the opening melody of Queen’s *Bohemian Rhapsody*, John Lennon’s *Imagine*, or Beethoven’s *9th Symphony* pops into your head, would you feel like the melody was pretty accurate? For most of us, the answer would be yes. Memory for relative pitch—the ability to accurately recall the distance from one tone to the next—is highly prevalent in the general population and is critical in both the interpretation of prosody in speech and of melody and harmony in song and, as such, is an adaptive and advanced cognitive ability (Plantinga & Trainor, 2005; Saffran, 2003).

However, despite your confidence about the melody, you would probably be less certain about the accuracy of the specific absolute pitches (i.e., whether your imagery is in the correct key). An estimated less than one in 10,000 of the general population possesses perfect absolute pitch (AP), the ability to name a perceived pitch with no reference tone (Deutsch, 2013). You are likely able to conjure a mental image of a tone, but for most of us, there is no ability to accurately and confidently assign a label to that tone.

Although most people raised in English-speaking, Western populations cannot name isolated absolute pitches, past research provides evidence for *implicit* AP. In previous studies, participants have shown a high level of consistency between multiple judgments of the same nonmusical stimulus (Deutsch et al., 1987); the ability to determine whether a learned canonical pitch (such as a dial tone or a familiar theme song) has been transposed (Schellenberg & Trehub, 2003; Smith & Schmuckler, 2008); and the ability to produce well-known melodies with high consistency between productions, implying a stable representation of pitch (Halpern, 1989).

Levitin (1994) demonstrated a particularly powerful example of this implicit absolute pitch in voluntarily recalled melodies. Participants were instructed to choose a song they knew well from a shelf of CDs, all of which had only one canonical recording (and therefore only one “correct” key), and were asked to imagine hearing the song in their heads and then to sing it out loud. Participants’ productions were compared to the original songs, and across two trials, 26% and 23% of participants, respectively, produced songs in the correct key, rounded to the nearest semitone (compared with 8.3% expected by chance). These results suggest that some form of absolute pitch is more common than previously thought and can be reliably elicited in a lab setting. Frieler and colleagues (2013) conducted the only known replication of this study across six different European labs, with 277 total participants producing two songs each, and found confirmation of the effect, although with a significantly smaller effect size than was reported in Levitin’s original experiment. In each trial, 15% of participants produced songs in the correct key, rounded to the nearest semitone (compared with 8.3% expected by chance).

One question that has not been addressed is whether this implicit representation of absolute pitch is uniquely part of the voluntary memory retrieval process or whether it extends to the involuntary retrieval process of musical memories, or *involuntary musical imagery* (INMI). Studies that compared involuntary autobiographical memory to voluntary autobiographical memory have found differences in both speed and detail of the retrieved experience, as well as an influence of (1) cue valence on the efficacy of the memory trigger, (2) differences in specificity between the two types of recall, (3) and a stronger influence of participant’s mood on recall accuracy in involuntary recall than in voluntary recall (Berntsen, 2010; Berntsen & Hall, 2004; Schlagman & Kvavilashvili, 2008). INMI is a type of musical imagery that some researchers have classified as a form of involuntary memory, specifically involuntary semantic memory (Kvavilashvili & Anthony, 2012; Kvavilashvili & Mandler, 2004). As such, it is possible that features of INMI differ from features of voluntarily recalled musical imagery.

Although some researchers have used INMI as a broader umbrella term that includes phenomena such as musical hallucinations and musical obsessions (Williams, 2015), in this paper we use the term to refer to the everyday mental experience of musical imagery playing, often on a loop, outside of conscious control—colloquially known as earworms. A survey of nearly 12,000 Finnish participants performed by Liikkanen in 2008 found that this ubiquitous human phenomenon is experienced by as much as 97.7% of the population at least once a month (with presumably similar rates found in Western industrialized populations around the world), with one study estimating that as much as 47% of people’s waking time, on average, is spent experiencing INMI (Floridou & Müllensiefen, 2015). Other foundational work in INMI research has proposed models of what causes INMI episodes to occur (Williamson et al., 2012), and provided grounded theory approaches to understanding the “form and function” of INMI episodes, including both vividness of imagery and people’s attitudes toward the experience (Williamson & Jilka, 2014).

Investigating the musical characteristics of INMI is challenging because these experiences happen spontaneously and at unpredictable times of the day. To avoid the pitfalls associated with retrospective judgments, researchers have implemented real-time reporting methods to capture the specific characteristics of INMI experiences, such as prevalence, triggers, and tempo (Bailes, 2006, 2015; Floridou & Müllensiefen, 2015; Jakubowski et al., 2015; McNally-Gagnon, 2016). In the diary method, participants report phenomena as they become aware of them. One study of note has used the diary method to examine the accuracy of the tempo of INMI. Jakubowski and colleagues (2015) gave participants an accelerometer on which they would tap the tempo of INMI episodes as they arose throughout their day and a paper journal to record the name of each tapped song. These recorded tempi were later compared to the tempo of the original song and found to be highly veridical; over three-quarters of reported INMI experiences were tapped within ± 15% of the original tempo. Taken in aggregate, the reported INMI tempi and original song tempi were highly correlated, *r* = 0.77, *p* < 0.001. This study provides proof of concept that musical details of INMI can be captured with real-time reporting methods. An unpublished PhD dissertation, which was recently brought to the authors’ attention, also used a diary method to provide evidence for the veridicality of tempo and pitch in INMI (McNally-Gagnon, 2016). Although the results of this PhD dissertation are intriguing, to date, no peer-reviewed study has directly investigated the pitch accuracy of INMI.

In sum, previous laboratory studies have found high veridicality of both tempo and pitch in voluntary musical imagery (VMI; Levitin, 1994; Levitin & Cook, 1996). Furthermore, a diary study by Jakubowski and colleagues (2015) found evidence for highly veridical tempo information in INMI, and follow-up work by Jakubowski and colleagues (2018) directly comparing the tempi of VMI and INMI collected through a diary method found that the two were highly positively correlated. It remains unknown whether the parallels between voluntary and involuntary recall of musical memory that have been observed in the domain of tempo extend to absolute pitch. Following similar logic to that proposed by Jakubowski and colleagues (2015), if the present study finds evidence that accurate recall for absolute pitch information is present within INMI, it would suggest that the parallels between voluntary and involuntary retrieval of musical memories extend to absolute pitch information. On the other hand, if veridical absolute pitch information is not present in INMI, it would suggest that the parallels between these two memory retrieval processes do not extend to absolute pitch, and perhaps other factors, such as participant mood or cue valence, are influencing the involuntary recall process (see Jakubowski et al., 2015).

In the present work, we apply the *experience sampling method* (ESM) to study the absolute pitch information present in INMI experiences (see Bailes, 2015; Floridou & Müllensiefen, 2015). In this method, participants are prompted by text messages at pseudorandom times throughout the day and asked to report on any INMI they are experiencing at the time of notification. We collected hummed and sung recordings of INMI experiences from participants and compared those fragments with the corresponding fragments of the original songs, whenever those songs had only a single canonical recorded version. Although our study does not directly compare productions of VMI with INMI, it does allow us to investigate whether INMI experiences preserve the absolute pitch of the original songs. If veridical absolute pitch is observed in INMI, it would add to the evidence of overlapping or parallel processes in recall of voluntary and involuntary musical memories; if veridical absolute pitch is not observed in INMI, it would suggest that either different retrieval mechanisms are in place or that additional factors such as participant mood or cue valence influence the involuntary recall process for absolute pitch information.

## Methods

### Participants

Our convenience sample consisted of 30 undergraduate students (26 women, four men) recruited from the University of California, Santa Cruz, psychology participant pool and by word of mouth. Participants’ age ranged from 18 to 27 years (*M* = 20.13, *SD* = 2.18). All participants were prescreened to ensure they spoke English, were located within the United States, and owned a smartphone capable of receiving SMS text messages and connecting to the internet. Participants were not screened on the basis of musical training or ability, although in a postexperiment survey, 25 out of 30 reported at least 1 year of formal training on a musical instrument. Importantly, all 30 participants self-reported that they do not have perfect pitch. The UC Santa Cruz Institutional Review Board approved the study, and all participants were compensated at the end of the two-week study period (estimated at approximately 5 h of active engagement) with a $75 Amazon gift card.

Our sample size was limited to 30 due to the cost of recruitment and the high level of commitment required from each individual participant (2 weeks of active participation). We did not conduct a power analysis because previous studies that examined pitch production used a different paradigm that is not comparable to ours. Whereas previous studies on voluntary musical imagery only required two productions per participant, our study allowed participants to produce up to 42 recordings each.

### Ethics statement

The study protocol was approved by the Institutional Review Board of the University of California, Santa Cruz. Written informed consent was obtained for all participants. Selected voice recordings are available in the corresponding OSF repository with participants’ written consent (https://osf.io/83b97/).

### Materials

The Experience Sampling Survey consisted of 41 items (see Appendix A), adapted and extended from Jakubowski et al. (2015), Bailes (2015), and Thayer (1989). The survey was built using the open-source SurveyJS framework (MIT License), implemented as a website hosted on Amazon Web Services (AWS), and participants were prompted via SMS text message.

The survey architecture was built using AWS and Twilio API. AWS CloudFront was used to deliver the experience sampling survey as a static webpage, with user validation and submission being handled with AWS Lambda functions. On the backend, we had a Node.js server running a cron job, which assigned pseudorandom times for participants to be contacted each day and sent out SMS notifications through the Twilio Programmable Messaging API.

### Procedure

After signing up for the study, participants were invited to attend a brief virtual onboarding session via Zoom with a research assistant. Participants were asked to provide informed consent and to complete a media release form. Participants then provided their cell phone numbers and were assigned a participant ID. The researcher then shared their screen, and participants were asked to open the survey on their phones and follow along as the researcher took the participant through a sample survey, clarifying any questions as necessary. Participants were then told to expect to begin receiving notifications in the next 1 to 3 days, with the 2-week study period beginning on the first day a notification was received. They were also instructed to only respond to notifications if they were able to complete the survey as soon as they noticed that they had received the text message; otherwise, participants were instructed to skip that survey and wait for the next one. For example, if they received the text message while they were driving, but their phone was in their pocket and they did not notice it until they parked, they were instructed that they should respond to that survey; on the other hand, if they saw the text message while they were driving and were therefore unable to respond immediately, they were instructed that they should skip that survey.

Survey notifications were delivered by SMS text message six times a day, at pseudorandom times between 9:00 AM and 9:00 PM (local time for each participant), every day for a period of 14 days. Each survey notification was separated by a minimum of 30 min from the previous one. The first question on each survey was, “At the time you received the notification, was there music playing in your head?” Participants were instructed during onboarding that this question was referring specifically to mental imagery that was not being voluntarily held in mind at the time of the notification. If participants answered “yes,” they were asked to record their INMI by singing into their phones, trying to match the pitch and tempo of their mental experience as accurately as possible. This was followed by 22 questions about the INMI occurrence and 19 questions about what the participant was doing at the time they received the notification, as well as their mood. If participants answered they were not experiencing an INMI, they answered only the 19 questions about their activities and mood.

The 14-day period was separated into a Record week and a No-Record week (the order of which was counterbalanced across participants). During No-Record weeks, the survey was identical to Record weeks, with the exception of skipping the step asking participants to record their INMI. We did this in order to ensure that the process of recording INMI experiences did not interfere with the frequency of reported INMI experiences. At the end of the 2 weeks, participants were contacted by email to complete a postexperiment survey via Qualtrics. This survey consisted of a demographics questionnaire and the Goldsmiths Musical Sophistication Index (Müllensiefen et al., 2014).

## Results

### Descriptives

In total, 2,520 surveys were delivered to participants, of which 1,928 responses were returned, for an overall compliance rate of 76.51%. Of the 1,928 responses, 980 (50.83%) were received during Record weeks, and 948 (49.17%) were received during No-Record weeks, revealing no difference, *t*(2518) =  − 1.507, *p* = 0.1328, *9*5% CI [− 0.0077, 0.0585]; this suggests that participants had no bias towards responding or not responding to a survey based on whether it was a Record or No-Record week. Individual participant compliance rates ranged from 22.62% to 100% (*M* = 76.63%, *SD* = 22.11%**)** over the 2-week study period.

Of the 1,928 responses, 462 indicated the occurrence of INMI, for an overall average INMI prevalence of 23.96%. Individual INMI prevalence ranged from 0% to 64.56% (*M* = 25.56%, *SD* = 18.09%). Only one participant in our sample did not report experiencing INMI at all during the study period.

Of the 462 INMI reports, 212 (45.59%) occurred during Record weeks, and the remaining 250 (54.31%) came from No-Record weeks. This difference in the proportion of INMI reports between conditions, although small, was statistically significant, *t*(1926) =  − 2.44, *p* = 0.015, 95% CI [− 0.0855, − 0.0093], *d* = 0.12. This suggests participants had a slight bias to respond “No” to the INMI question during Record weeks, possibly to avoid having to record their INMI experiences through singing.

Participants also completed the Goldsmiths Musical Sophistication Index (GMSI), and these results are summarized in Table 1, found in Appendix B. GMSI questions are scored from 1 to 7 and divided into subscales. Of particular interest are the scores on the Musical Training subscale (*M* = 3.41, *SD* = 1.44, range: [1.00, 5.86]) and the General Sophistication subscale (*M* = 4.13, *SD* = 0.91, range: [2.17, 5.67]), indicating that our sample is not highly musically trained, and is of average general musical sophistication.

### Preparing the data for pitch analysis

To analyze the relationship between the pitch of the recorded INMIs and the pitch of the original songs, we had to discard 67 of the 212 recordings from further analysis: 21 recordings were corrupted or missing; 40 recordings were unidentifiable (or the segment of the song from which the recording came could not be resolved); and finally, six recordings were of songs that have been recorded in multiple different keys (and the exact version was not specified by the participant in the follow-up survey). This left 145 recordings to potentially be included in the following pitch analyses; however, based on intercoder agreement, this number was reduced to 132.

### Pitch analysis

For each recording, we used a numerical optimization technique to estimate the difference in key between the original song fragment and the participant’s recording. For each INMI recording, research assistants began by finding the part in the original song corresponding to the participant’s recording. They then estimated and transcribed the discrete pitches of the entire song fragment to the nearest semitone, both for the participant’s recording and the corresponding fragment from the original song. Fragments ranged in length from four notes to 202 notes (mean = 37 notes, *SD* = 32.3). Participant productions were then octave-corrected to match the same octave as the original song fragment. Using an error minimization algorithm we identified the pitch offset in integer semitones that minimized the mean absolute error (MAE) between the recording and the original song (see Appendix C for a detailed description of this process). This allowed us to estimate the error of each participant-produced fragment as distance in semitones from the original. Unlike previous studies (Frieler et al., 2013; Levitin, 1994) who based the analysis on the first produced note, we based it on the entire song fragment. Because participants were not given a chance to rerecord their INMI to account for perceived production errors, we reasoned that the entire song fragment would produce more reliable results than simply the first note. This also accommodates for the possibility that participants changed keys midproduction, with the resulting score representing the prevailing pitch error across the full fragment. Selected examples of participants’ recordings, along with the corresponding original song fragments, are available online (https://osf.io/83b97/).

#### Intercoder agreement

Each pair of song fragments (the participant’s recording and the corresponding segment of the original song) was transcribed twice, in separate passes by independent coders. After the first two passes, 103 out of the 145 pairs of recordings (71.03%) had full agreement between coders. Of the 42 pairs with disagreement, 24 (57.14%) disagreed by only 1 semitone. All 42 pairs with disagreement were given a tiebreaker pass, with a third independent coder retranscribing the first 15 notes of each pair. After this tiebreaker pass, 29 of these 42 pairs were in agreement between two of the three passes (this 2 out of 3 agreement of error was used for the following analysis), and the remaining 13 recording pairs were discarded. At the end of this process, six participants were left with no usable recordings. This left 132 recordings from 24 participants for any analyses involving pitch.

### Pitch results

#### Pitch error

To test whether pitch errors were significantly smaller than chance, we made the same assumptions regarding the most reasonable null hypothesis as Levitin (1994): that participants would have no bias towards producing their INMI in any particular key, and the null distribution would be uniform.

**Fig. 1:**
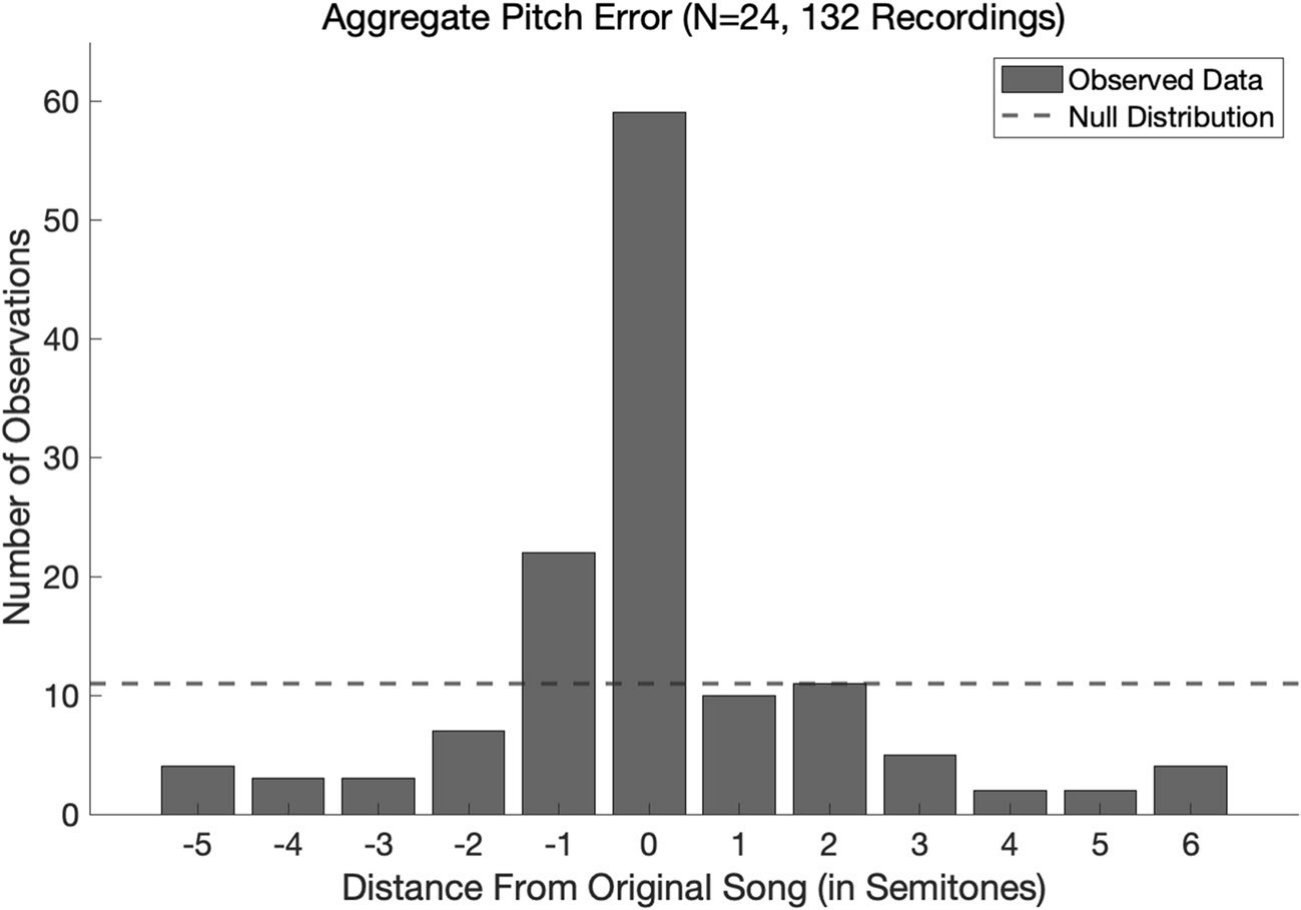
Aggregate pitch error. *Note.* This figure shows the aggregate pitch error across all recordings in discretized semitones, adjusted for octave. The dashed line indicates the distribution of pitch errors that would be expected by chance

The results, shown in Fig. 1***,*** show a highly nonuniform distribution with a large central peak. Overall, 59 out of 132 recordings (44.70%) had an error of 0 semitones (i.e., were produced in the exact same key as the original, to the nearest semitone). A binomial test revealed this to be highly significantly higher than the 8.33% expected by chance, *p* < 0.001. Allowing for an error of up to ± 1 semitone, the number of on-key recordings rose to 91 out of 132 (or 68.94%), compared with the 25% expected by chance, *p* < 0.001.

Because our data are octave-corrected pitch classes, which is a circular modality, Rayleigh’s test of uniformity is the most appropriate analysis (Krumhansl, 1990; Levitin, 1994; Shepard, 1964). We are able to reject the null hypothesis of uniformity in favor of the alternative hypothesis that the data fit a circular normal distribution centered around a mean error of 0 semitones, *r* = 0.6367, *p* < 0.001.

#### Investigating a potential influence of recency

**Fig. 2:**
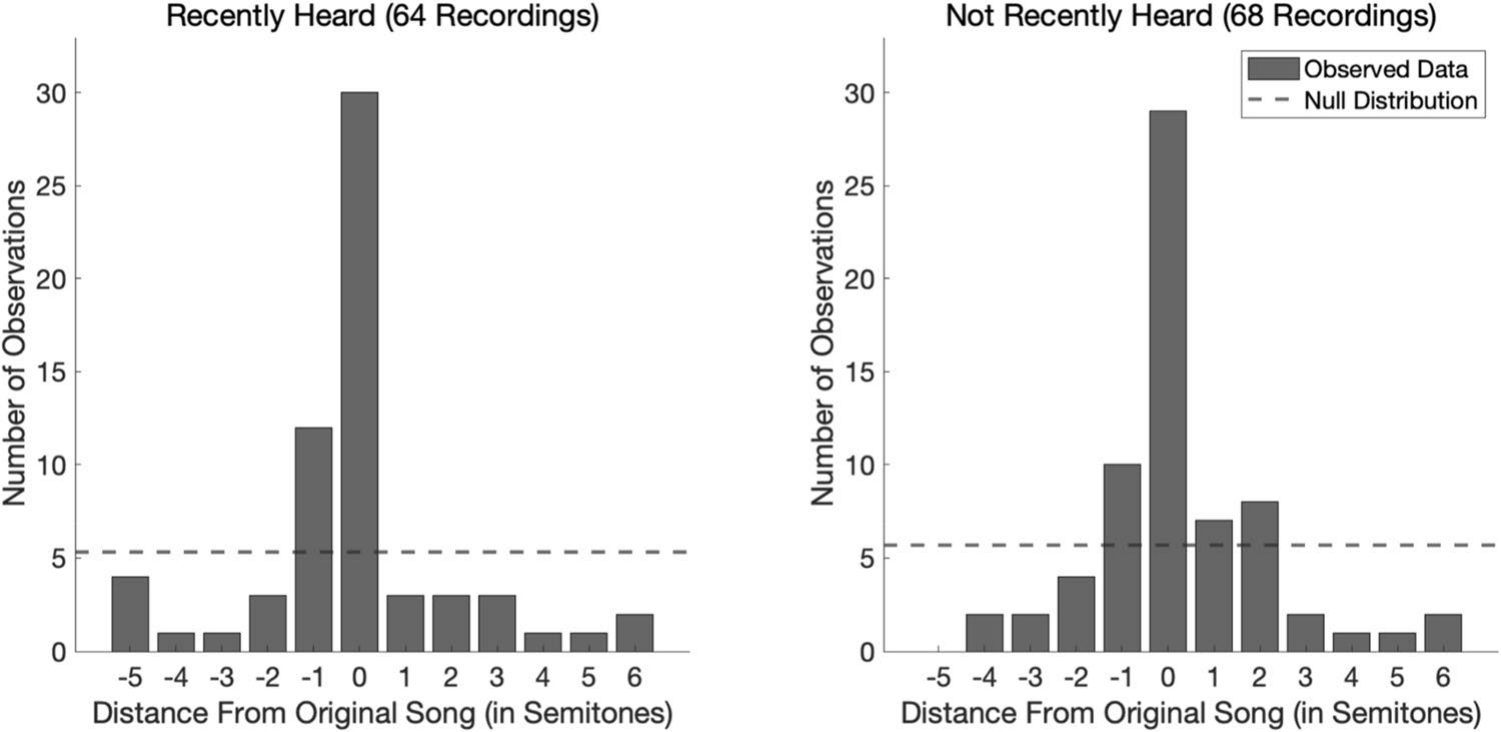
Investigating an effect of recency. *Note.* Distributions of pitch error for songs that were reported as recently heard (left) versus songs that were not reported as recently heard (right). The dashed lines represent the null distributions

One of the survey questions asked participants to report how their INMI was triggered (if they knew). Because the most commonly reported INMI trigger was “I recently heard a recording of this song” (64 out of 132 responses, or 48.48%), it is possible that our results were driven by INMI experiences that were accurate because of the persistence in short-term memory of a recent reference tone. However, when comparing the absolute pitch error of INMI triggered by hearing a recent recording (*N*_*1*_ = 64, *M*_*1*_ = 1.31, *SD*_*1*_ = 1.75) with the absolute pitch error of all other INMI experiences (*N*_*2*_ = 68, *M*_*2*_ = 1.21, *SD*_*2*_ = 1.48) using an independent-samples *t* test, we found no evidence of a difference, *t*(130) = 0.38, *p* = 0.71, 95% CI [− 0.4513, 0.6645] (see Fig. 2). This suggests that our results are not driven by short-term memory for reference pitches, and that accurate pitch information in INMI is instead represented in long-term memory.

#### Participant-wise analysis of absolute pitch error

When presenting the data in aggregate, it is possible that the central spike is being driven by a relatively small number of participants who produced a large number of accurate recordings. To ensure our results were not being driven by a small handful of highly accurate participants, we performed a participant-level analysis. In order to calculate each participant’s error, we considered the absolute error of each INMI so that positive and negative errors do not cancel out. Once converted to absolute values, there is only one way to be off by 0 semitones, two ways to be off by 1, 2, 3, 4, or 5 semitones, and only one way to be off by 6 semitones (collapsing across octaves). Therefore, an average absolute error of 3 semitones across all of a given participant’s productions would be expected by chance.

**Fig. 3:**
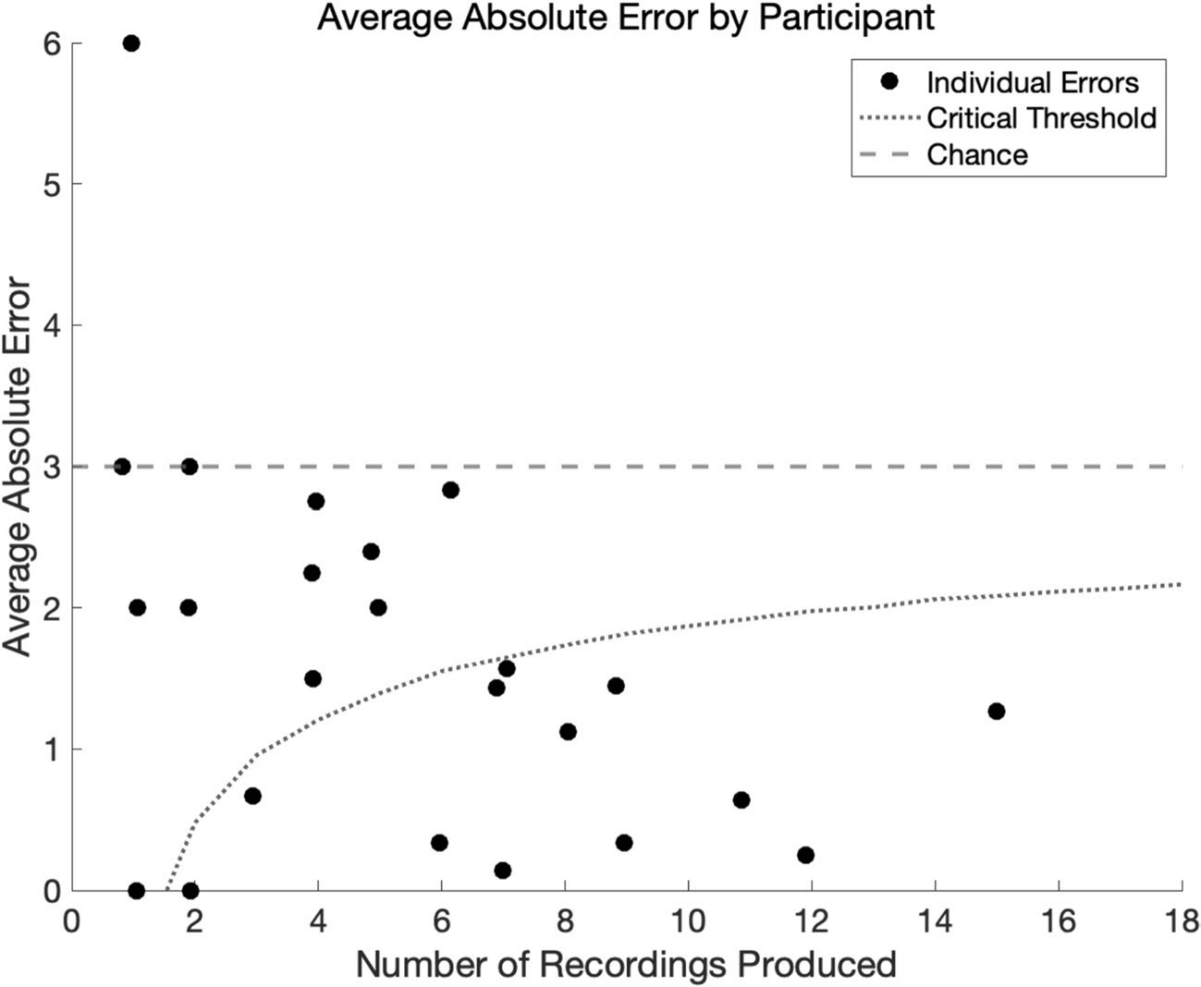
Average absolute error by participant. *Note.* Critical thresholds for absolute error based on the number of recordings produced. As the number of recordings produced increases, the critical threshold approaches numerical chance (an average absolute error of 3). The dashed line represents an average absolute error of 3. Points below the curved dotted line represent participants whose average absolute error was significantly smaller than 3 (*p* < .05)

Of the 24 participants included in the pitch analysis, 21 performed numerically better than chance, with average absolute errors of less than 3 semitones (binomial test, *p* < 0.001; see Fig. 3). However, the statistical significance of each individual participant’s average absolute error is dependent on the number of recordings they produced. To illustrate this, a participant who produced only one recording could not reasonably be said to be statistically better than chance even with an error of 0 semitones, since this could happen by chance 1 out of 12 times (or 8.33%, above the significance level of 0.05). Therefore, in order to determine the statistical cutoff for significance as a function of the number of recordings produced, we simulated 10,000 samples from the null distribution of absolute errors for each number of possible productions (1 through 20). The critical threshold for each *N* (where *N* = the number of productions) was calculated as the mean of each simulated distribution minus two times the standard deviation of that simulated distribution. This significance curve is seen superimposed on top of the individual results in Fig. 3.

Using these corrected critical thresholds for statistical significance, we now observe that 12 out of 24 participants (50%) produced recordings that were significantly more accurate than would be expected by chance. Importantly, we do not see any trend toward multiple data points in the lower right quadrant of the graph, so the aggregate results do not appear to be driven by a small number of highly accurate and highly productive participants.

### Additional factors that may influence pitch accuracy

#### Investigating a possible influence of voluntary recall

Participants were also asked to report, on a 7-point Likert scale, whether they had started their musical imagery on purpose or not (with a 1 being *not at all on purpose* and a 7 being *completely on purpose*). Although it is not necessarily the case that imagery that began voluntarily cannot then continue on involuntarily, there may still be an effect of that initial voluntary recall on pitch accuracy. An initial correlational analysis found no relationship between this self-report measure and signed pitch accuracy (*r* =  − 0.11, *p* = 0.194). We then considered separating the recordings into those with imagery that was begun voluntarily, and those with imagery that was begun involuntarily. Of the 132 participant recordings, 19 had a score of 4 or higher on this self-report scale, and were thus classified as being initially recalled voluntarily. A Rayleigh’s test of uniformity was performed on just the 113 “involuntary” recordings with the “voluntary” recordings removed, and we are still able to reject the null hypothesis of uniformity in favor of the alternative hypothesis that the data fit a circular normal distribution centered around a mean error of 0 semitones (*r* = 0.63, *p* < 0.001). Likewise, a Rayleigh’s test of uniformity performed on only the 19 “voluntary” recordings also allows us to reject the null hypothesis of uniformity in favor of the alternative hypothesis that the data fit a circular normal distribution centered around a mean error of 0 semitones, *r* = 0.7123, *p* < 0.001. Finally, an independent-samples *t* test was unable to detect a difference between the two sets of recordings, *t*(130) =  − 0.8138, *p* = 0.417, 95% CI [− 1.42, 0.59]. Therefore, we can rule out the possibility that our results are driven by voluntarily produced musically imagery.

#### Investigating a relationship between pitch error and tempo error

It is possible that participants have a learned relationship between sped-up tempo and higher pitch (as well as slowed-down tempo and lower pitch), from general exposure to this effect in digital audio manipulation, which is often seen in TikTok videos and other social media, or with analog playback devices such as turntables and tape players. We tested whether there was a correlation between signed tempo error and signed pitch error. In order to perform this analysis, each recording was analyzed for tempo by independent coders. The tempo was determined using a publicly available tap-to-BPM (beats per minute) web app (Reel, 2019). After two independent coders tapped the tempo, the mean error for each was computed by comparing the tapped tempo of the original song and the participant’s recording. If the mean error calculated by each coder differed by less than 10%, the average of these two errors was taken. If the mean error calculated by each coder differed by more than 10%, a third coder assessed the tempo, and the mean was taken between the two closest matched tempi. In four cases, none of the three coding passes were within 10% of any other pass and were thus discarded. This left 128 recordings for any analyses involving tempo.

We found a small significant correlation between signed pitch error and signed tempo error, *r* = 0.1806, *p* = 0.04. Although the effect is small, this is suggestive of a common mechanism by which earworms may be accelerated or decelerated relative to the original recording, which affects both pitch and tempo simultaneously.

We also looked at the relationship between absolute tempo error and absolute pitch error, since it is possible that participants with stronger musical ability—and therefore a stronger internal sense of tempo and rhythm—might also have more accurate pitch representation and production. However, the relationship between absolute tempo error and absolute pitch error did not reach statistical significance, *r* = 0.1476, *p* = 0.096.

#### Experience sampling variables

In order to analyze what factors may influence pitch accuracy for any given INMI experience, we conducted a stepwise regression using absolute error as the dependent measure, and responses from the Experience Sampling Questionnaire as the predictor variables. The predictor variables included in the model were: the vividness of the imagery (subdivided into the vividness of melody, instruments, and voice); meta-judgments of how accurate the participant thought their imagery’s pitch was; and familiarity with the song. The only significant predictor of pitch accuracy for any given INMI experience in this model is the vividness of the instrumental aspect of the imagery (*R*^2^ = 0.0392, *p* = 0.0228), although this result should be interpreted with a degree of caution due to the large number of comparisons being made.

#### Accuracy of voluntary versus involuntary imagery

The question remains as to whether there is a difference between the pitch accuracy of voluntarily recalled musical imagery and spontaneously occurring INMI. Although our study did not directly compare INMI to VMI, we refer to previous literature on absolute pitch accuracy in VMI to make an exploratory comparison. On visual inspection of the distributions of error in Levitin’s (1994) and Frieler et al.’s (2013) datasets, our data appear to be even more centrally peaked. If this were true, it would suggest that voluntary recall of musical imagery may be more error-prone with respect to pitch than spontaneously occurring INMI.

To test this, we performed a simulation that randomly sampled one data point from each of our participants in order to closely approximate Levitin’s methods, in which each trial consisted of a participant singing a single song. We then calculated the average absolute error of the resulting distributions and compared it to the average absolute error of a distribution of an equal number of randomly sampled data points from Levitin’s Trial 1 data, and the two errors were compared. We limited this comparison to Trial 1 only because, as Levitin suggests, it is possible that Trial 2 errors are artificially high due to the influence of the song produced in Trial 1. After 1 million simulations, the proportion of samples from our data with a smaller absolute error than Levitin’s was 0.7178; therefore, despite the average error being numerically smaller in our study, we cannot reject the null hypothesis based on an alpha of 0.05 that the data from the two studies are similarly centrally peaked.

The replication of Levitin’s (1994) study by Frieler and colleagues (2013), however, found a substantially smaller effect across a much larger pool of participants (*N*_Levitin_ = 46, *N*_Frieler_ = 277). When we perform a similar simulation to compare our data with Frieler and colleagues’ data, we find that the proportion of samples from our data with a smaller absolute error than Frieler’s Trial 1 data is 0.9783. At an alpha level of 0.05, we are able to reject the null hypothesis that the two datasets are equally distributed, in favor of the alternative hypothesis that our data are more centrally peaked around an average error of 0. Thus, we have preliminary evidence that involuntary musical imagery may be even more accurate with respect to absolute pitch than voluntarily produced musical imagery. However, it should be noted that these analyses are exploratory and should be interpreted with caution, as there are substantial methodological differences between our experience sampling study and these laboratory studies. For example, in the laboratory studies, participants chose songs from a list, whereas in our study participants produced songs that came freely to mind. It is possible that our participants had more familiarity with the songs they produced, and this familiarity is the cause of any improved absolute pitch accuracy. Future work should directly compare absolute pitch accuracy in voluntary and involuntary musical imagery to determine if absolute pitch accuracy differs between VMI and INMI when factors such as familiarity are held constant (see Jakubowski et al., 2018).

### Consideration of an alternative null hypothesis based on vocal range

There is an alternative hypothesis that would explain the pitch accuracy results we report here. Perhaps one of the reasons that popular music is popular is because it falls within the comfortable singing range of most listeners. If that were true, it would perhaps also be true that participants simply produce recordings of their INMI experiences centered around the midpoint of their comfortable singing range, which might correspond (on average) with the correct key of many popular songs.

**Fig. 4:**
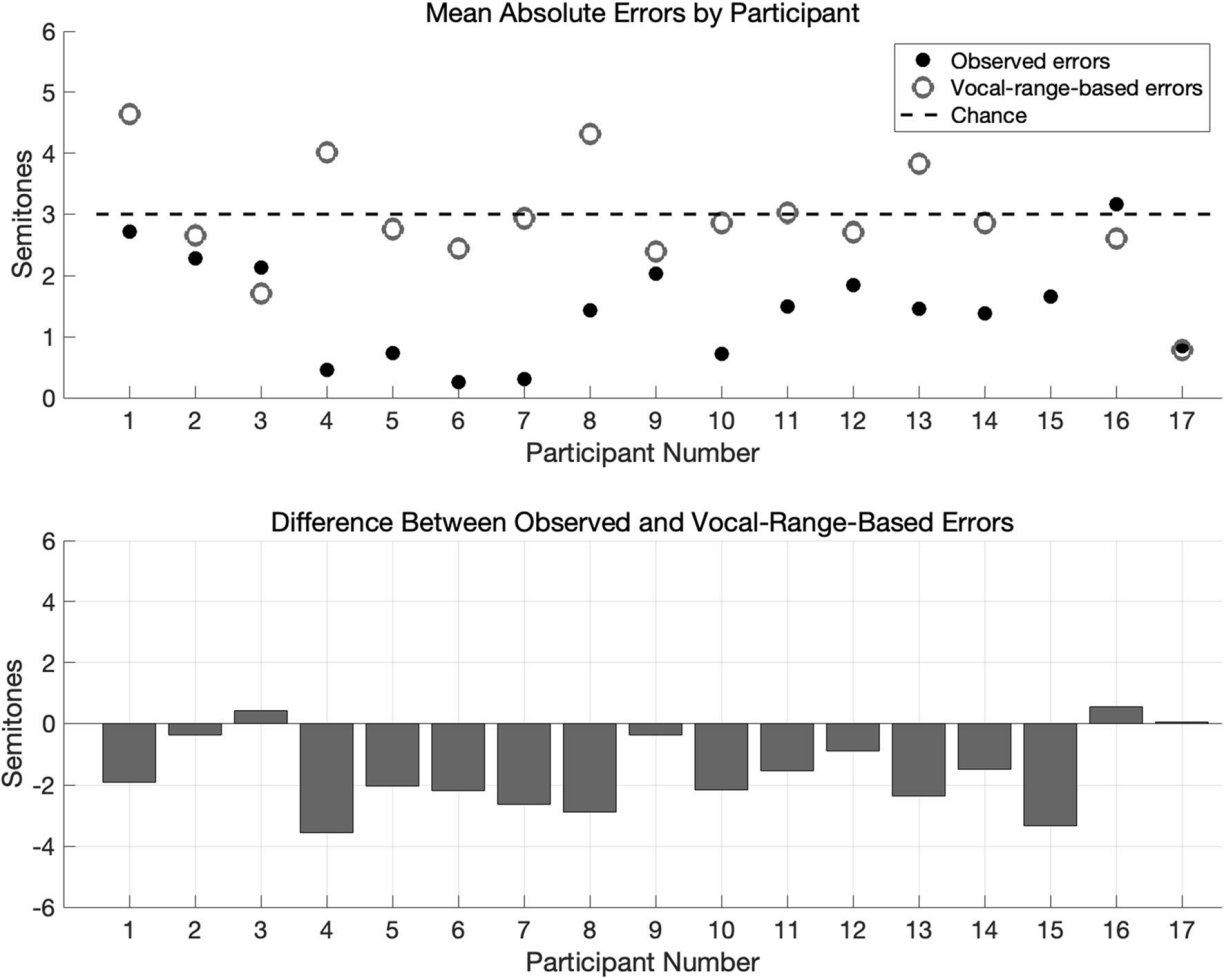
Investigating an alternative hypothesis of vocal range. *Note.* Top: Observed mean absolute errors by participants who produced at least 3 recordings shown in solid dots. Gray circles indicate mean absolute errors predicted by each participant’s estimated vocal range. The dashed line represents chance. Bottom: Difference between observed mean absolute errors and absolute error predicted by each participant’s estimated vocal range

In order to rule out this explanation, we estimated each participant’s vocal range by considering the lowest and highest note produced by each participant across all of their productions—and for the purpose of this analysis, we included only the 17 participants with at least three productions, since the vocal range estimate would be unreliable for participants with only one or two productions. Based on this analysis, the mean estimated vocal range across participants was 19.25 semitones (*SD* = 9.15, range: [9, 34]), roughly one and a half octaves—comparable to the typical vocal range of nonmusicians (Moore, 1991). The midpoint of each participant’s estimated range then served as the default central pitch for productions by that participant under the “vocal range hypothesis.” We measured the expected distribution of pitch errors if participants had always made productions centered on their central pitch. For example, if a participant’s estimated vocal range went from A3 to E4, their central pitch would be defined as G3. Their expected error would be computed by comparing the original song’s key to the key that would result from a production of this song with G3 as the central pitch. We then compared this distribution of expected errors with the observed distribution of pitch errors. Under the “vocal range hypothesis,” the expected average absolute error across participants would be 3.03 semitones (similar to the chance level expected by a uniform distribution). In contrast, the observed average absolute error was 1.47 semitones—significantly smaller than the error expected under the vocal range hypothesis, *t*(19) =  − 5.06, *p* < 0.001, 95% CI [− 2.22, -0.91] (see Fig. 4). Thus, the alternative hypothesis that participants were simply centering their productions around the midpoint of their vocal range does not explain our results.

We also considered the possibility that a smaller vocal range could constrain a participant’s production and result in a larger absolute pitch error. To test this, we conducted a correlational analysis between participants’ observed vocal range (in semitones) and their average absolute pitch error. This analysis revealed a marginally significant negative correlation (*r* =  − 0.47, *p* = 0.056), suggesting that participants with larger vocal ranges did tend to produce smaller pitch errors. We note that our estimate of vocal range is limited, especially for participants who produced very few songs. Indeed, these estimates of participants’ vocal range were positively correlated with the number of songs they produced (*r* = 0.81, *p* < 0.0001); however, this did not statistically significantly predict these participants’ average absolute error (*r* =  − 0.43, *p* = 0.084). Another possibility is that participants with more productions had multiple opportunities to produce each song in the right key. However, our data showed very few instances of repeated songs (only four participants produced at least one repetition, and only seven repetitions were found in total across the 122 productions included in this analysis). Therefore, neither participants’ estimated vocal range nor their number of productions can fully account for our results.

## Discussion

The present study provides novel evidence that the general population has remarkably accurate memory for the absolute pitch of songs, as measured in their spontaneously occurring musical imagery. Of the 132 produced recordings that we could analyze for pitch accuracy, 59 (44.70%) had an error of 0 semitones, and 91 (68.94%) had an error within 1 semitone, much higher than would be expected by chance. At the individual participant level, 21 out of 24 participants who produced recordings had an average absolute error smaller than the 3 semitones that would be expected by chance, and errors were statistically significantly smaller than chance for 12 out of 24 participants.

Although none of our participants reported having absolute pitch, 50% of them showed a statistically significantly better-than-chance pitch memory for unprompted, involuntary musical imagery. This finding reveals that absolute pitch memory is present in natural settings, not just in a lab setting with voluntarily recalled melodies chosen specifically for their high level of familiarity, as shown by Levitin (1994) and Frieler et al. (2013). In fact, because our participants were not trained singers, some may have experienced difficulty in accurately producing the tones they were hearing in their heads during their INMI experiences. Therefore, it is possible that participants’ internal pitch memory is even more accurate than our methods are able to capture (see Levitin, 1994; Takeuchi & Hulsi, 1993). Although it is possible that memory errors and production errors could cancel out (producing accidentally veridical pitch), the chance of this accounting for our results is extremely low.

### Implications of absolute pitch in everyday INMI experiences

Our finding of accurate absolute pitch recall in participants’ everyday INMI experiences adds novel evidence supporting strong parallels between voluntary and involuntary recall of musical memories. As previously stated, laboratory research has found high veridicality of both tempo and pitch in voluntary musical imagery (Frieler et al., 2013; Levitin, 1994; Levitin & Cook, 1996). A diary study by Jakubowski and colleagues (2015) found evidence for veridical tempo information in INMI, with follow up work by Jakubowski and colleagues (2018) that directly compared the tempi of VMI produced in the lab with INMI collected through a diary method finding that the two were highly positively correlated. Our results provide novel evidence that these observed parallels between voluntary and involuntary recall of musical memory in the domain of tempo extend to absolute pitch information. A follow-up study could expand on our results by bringing participants who participated in an experience sampling study back into the lab and asking them to voluntarily produce a single song that they reported as INMI during the experience sampling period (see Jakubowski et al., 2018). This would allow for a direct comparison, within participants, of both the consistency of pitch memory for the same song, as well as the relative accuracy of pitch in involuntary versus voluntary music recall.

### Methodological considerations: Experience sampling versus diary method

It is worth noting here that, although we have mostly grouped the experience sampling method (ESM) and diary studies under the same umbrella of “real-time reporting methods,” there are differences between the two methodologies that are worth examining when planning future studies. In a diary method, as used by the Jakubowski and colleagues (2018) study and the unpublished PhD dissertation by McNally-Gagnon (2016), participants are asked to report INMI throughout the day as they become aware of them, with no upper limit on the number of reports per day; in contrast, the ESM’s explicit prompts may increase the salience of INMI experiences that were already occurring just outside of conscious awareness, a set number of times per day. There may, therefore, be differences in the features of INMI experiences reported in the two methods; for example, while INMI experiences reported under the diary method may be more vivid and salient, reaching the threshold of conscious awareness required for self-prompted reporting, INMI experiences reported under the ESM may be less consciously accessible until the time of prompting. If characteristics of INMI differ depending on how consciously available they are, researchers may be tapping into different phenomena when using the two different methods. Although these potential differences should be considered in future experiments, both methods are undoubtedly useful for capturing spontaneous and involuntary phenomena.

## Limitations

The present study is, to our knowledge, the first attempt at using experience sampling and participant-sung recordings to gather information on the pitch content of INMI experiences. In gathering, discussing, and analyzing these data, we have identified a number of limitations to our design that could be addressed and improved upon in future research.

Due to the relatively small sample size, we are not able to draw further conclusions regarding individual differences or how our results would generalize to a broader population. Additionally, we did not screen based on whether participants were raised as native speakers of a tonal language, which could have implications for their representation of absolute pitch. Our recruitment materials included the phrase “musical imagery in everyday life,” so participants with a higher-than-average rate of INMI or musical ability may have self-selected into the study. Future research should be more cautious to avoid this potential bias in sample selection.

Participants were given the option to report whether their INMI experiences had been triggered by recently hearing the song, but we did not ask *how* recently that trigger had occurred. Although we saw no difference in pitch error between these INMI experiences triggered by recently heard songs and all other INMI experiences, there may still be a difference that our methods were not sensitive enough to detect. For instance, a song that was heard 1 min ago may trigger a more accurate INMI experience than one that was heard an hour ago, even though both may be classified as “recent.” Future research using this paradigm should collect more fine-grained information about how recently the song was heard in order to further investigate a potential relationship between the recency of hearing the song and the pitch accuracy of the earworm.

The use of song recall as a method of probing absolute pitch representation relies on the song being recalled having only one canonical version. With the ubiquity of social media platforms like TikTok that often present sped-up or slowed-down versions of popular songs, users may be getting exposed to alternate-pitch versions of songs that previously had only one canonical version. Future studies that rely on song recall to probe absolute pitch representations must be attentive to this limitation by including questions about the exact source of the musical memory, specifically whether it is of the original recording or an altered version.

## Conclusion

The present study provides novel evidence that for a large proportion of the population, spontaneously occurring earworms preserve the absolute pitch of the original song. In our sample, 21 out of 24 participants had an average absolute pitch error smaller than the 3 semitones that would be expected by chance, and for 12 out of 24 participants, that error was statistically significantly smaller than 3 semitones. Overall, 44.70% of INMI experiences observed across all participants had an error of 0 semitones, and 68.94% had an error within 1 semitone. The lack of difference between the error in INMI experiences that were reported to be triggered by recently hearing the song versus not suggests that INMI experiences retain absolute pitch information even across large lapses in time. These findings provide the first evidence that absolute pitch memory is automatic and does not require deliberate effort. Instead, accurate absolute pitch memory is revealed in spontaneously occurring musical imagery.

## Supplementary information

Below is the link to the electronic supplementary material.

Supplementary file1 (DOCX 35 KB)

## Data Availability

The full raw data have not been made available on a permanent third-party archive because our institutional review board ruled that we could not post the data; requests for the deidentified data can be sent to the corresponding author, matt.evans@ucsc.edu. The complete questionnaires and detailed explanations of data analysis methods are included in the appendices of this article. Three example sample participant recordings, along with the corresponding original song fragments, are available online (https://osf.io/83b97/).

## Code availability

Requests for the experience sampling code or analysis code should be directed to the corresponding author, magievan@ucsc.edu.

## Declarations

### Open practices statement

The study reported in this article was not preregistered. The full raw data have not been made available on a permanent third-party archive because our institutional review board ruled that we could not post the data; requests for the deidentified data can be sent to the corresponding author. The complete questionnaires and detailed explanations of data analysis methods are included in the appendices of this article. Three example sample participant recordings, along with the corresponding original song fragments, are available online (https://osf.io/83b97/).
