# Supplementary material for: Absolute pitch in involuntary musical imagery
Source: Atten Percept Psychophys. 2024 Aug 12;86(6):2124–35. doi: 10.3758/s13414-024-02936-0 (PMC11411011; doi:10.3758/s13414-024-02936-0)

**Appendix A**

**Earworm ES Prescreening Interview Questions**

**(Participants must answer yes to all questions to be eligible)**

1. Do you speak English? *(English need not be first language)*
2. Are you physically located within the United States (and will be for the duration of the two week study period)?
3. Do you own a smartphone capable of receiving SMS text messages and connecting to the internet? *(submitting each survey does require the use of a very small amount of data, <2 MB each)*

**Experience Sampling Questionnaire (delivered 6 times daily)**

1. At the time you received the notification, was there music playing in your head? (Note: if there was music out loud at the time you received the notification, please answer no) *Yes/No*
   1. If no:
      1. Skip to **2**
   2. If yes:
      1. Please record it. Do your best to accurately record what you were hearing in your head, including lyrics if applicable. Keep as close as you can to the pitch and tempo of your mental experience, as well as the duration.
         1. How accurately did the pitch and tempo of your recording match what you were hearing in your head?: *1 (not at all) - 5 (exactly the same)*
         2. Do you think your imagery is in the same key as the original song?: *Yes, No, Unsure, or N/A*
      2. Approximately how long have you had this particular song in your head?: _______(in minutes)
      3. Please describe how vivid your mental image of the following musical elements was:
         1. *Melody: 1(absent) - 7(very vivid)*
         2. *Lyrics (if applicable): 1(absent) - 7(very vivid)*
         3. *Instruments: 1(absent) - 7(very vivid)*
         4. *Voice: 1(absent) - 7(very vivid)*
         5. *Tempo/Rhythm: 1(absent) - 7(very vivid)*
      4. Lyrics of the part of the song that was in your head-- Please list AT LEAST THE FIRST AND LAST PART of the lyrics: ___________________
      5. How much do you agree with the following statement: This part of the song was looping in my mind  *1(strongly disagree) - 7 (strongly agree)*
      6. Was the music your own composition? *Yes/No*
      7. Name of song (if known): ________________
      8. Performer of song (if known): ________________
      9. How well do you know this song?: *1 (not well at all) - 7 (very well)*
      10. How much do you agree with the following statement? I made the music in my mind start playing on purpose: *1 (strongly disagree) - 7 (strongly agree)*
      11. Describe how you felt about the experience of hearing this song in your head: *1 (very annoyed by it) - 7 (really enjoy it)*
      12. How much do you like the song itself?: *1 (strongly dislike) - 7 (strongly like)*
      13. This musical imagery was distracting: *1 (strongly disagree) - 7 (strongly agree)*
      14. Have you experienced and recorded this particular segment of music as an earworm already during your participation in this study? *Yes/No*
      15. How frequently is this particular song or segment of music an earworm for you?
          1. *Multiple times a day*
          2. *Once a day*
          3. *Once a week*
          4. *A few times a week*
          5. *Once a month*
          6. *A few times a month*
          7. *This is the first time I’ve had this earworm*
      16. Please check as many boxes below as are applicable indicating why you might have been hearing this particular music in your head:
          1. *I have no idea why this song came into my head*
          2. *I recently heard a recording of this song*
          3. *I recently heard a friend or stranger singing, humming, or whistling this song*
          4. *I recently performed this song*
          5. *A person word, sound, or other occurrence in my environment reminded me of this song*
          6. *I was thinking about an event from my past and it reminded me of this song*
          7. *I was thinking about a future event and related it to this song*
          8. *Other (please explain)*
      17. Please select the best description of your experience:
          1. *I was hearing a complete song*
          2. *I was hearing a fragment*
          3. *Other ________________*
      18. Were you alone when you recorded this segment? *(yes or no)*
   3. Please check any of the following that describe what you were doing at the time you received the notification (check all that apply):
      1. *Walking*
      2. *Running*
      3. *Cycling*
      4. *Sitting*
      5. *Watching Television*
      6. *Reading*
      7. *Writing*
      8. *Working (physical)*
      9. *Working (academic)*
      10. *Driving*
      11. *Cleaning*
      12. *Cooking*
      13. *Using social media*
      14. *Daydreaming*
      15. *Other ___________________*
   4. How much do you agree with the following statement? My mind was wandering at the time I received the notification: *1 (strongly disagree) - 7 (strongly agree)*
   5. Mood questions
      1. For each pair of moods, make a selection that mostly describes the way you were feeling when you received the notification. (*Very - Quite - Somewhat - Neither - Somewhat - Quite - Very*)
         1. Alert - Drowsy
         2. Happy - Sad
         3. Lonely - Connected
         4. Energetic - Tired
         5. Involved - Detached
         6. Tense - Relaxed
         7. Interested - Bored
   6. Arousal questions
      1. Each of the following words describes feelings or mood. Please use the rating scale next to each word to describe your feelings at the moment you received the notification. (1 = *definitely do not feel - 10 = definitely feel*)
         1. Active
         2. Jittery
         3. Calm
         4. At-rest
         5. Lively
         6. Quiet

**Appendix B**

**Goldsmiths Musical Sophistication Index - Descriptive Results**

| Participant | Age | Gender | Active Engagement | Perceptual Ability | Musical Training | Singing Ability | Emotionality | General Sophistication |
| --- | --- | --- | --- | --- | --- | --- | --- | --- |
| 1 | 20 | Female | 2.44 | 3.44 | 1.86 | 2.86 | 5.00 | 2.17 |
| 2 | 23 | Female | 2.89 | 5.11 | 2.71 | 1.71 | 5.00 | 2.67 |
| 3 | 19 | Female | 2.67 | 4.78 | 4.43 | 4.14 | 4.67 | 4.33 |
| 4 | 22 | Female | 3.56 | 4.22 | 1.57 | 3.00 | 6.17 | 2.94 |
| 5 | 21 | Female | 5.67 | 5.22 | 1.00 | 5.43 | 6.00 | 4.11 |
| 6 | 18 | Female | 5.56 | 6.33 | 5.43 | 4.71 | 6.50 | 5.67 |
| 7 | 20 | Female | 5.00 | 6.44 | 5.29 | 5.14 | 6.00 | 5.33 |
| 8 | 18 | Female | 5.56 | 5.11 | 2.29 | 4.29 | 6.00 | 4.28 |
| 9 | 24 | Male | 5.78 | 4.67 | 4.14 | 5.29 | 5.00 | 5.22 |
| 10 | 18 | Female | 3.78 | 4.44 | 2.57 | 3.14 | 4.83 | 3.06 |
| 11 | 18 | Female | 4.11 | 4.56 | 3.14 | 4.29 | 4.67 | 3.78 |
| 12 | 20 | Female | 4.33 | 4.44 | 4.86 | 3.71 | 5.67 | 4.06 |
| 13 | 21 | Female | 2.89 | 4.22 | 1.00 | 3.86 | 4.67 | 2.89 |
| 14 | 27 | Male | 5.11 | 5.44 | 5.86 | 4.29 | 5.33 | 5.17 |
| 15 | 21 | Female | 2.00 | 4.78 | 4.29 | 5.43 | 4.33 | 4.22 |
| 16 | 18 | Female | 4.11 | 5.00 | 3.29 | 5.57 | 5.67 | 4.50 |
| 17 | 18 | Female | 3.89 | 5.44 | 4.43 | 4.71 | 5.50 | 4.61 |
| 18 | 21 | Female | 4.56 | 4.78 | 4.71 | 5.00 | 5.33 | 4.72 |
| 19 | 18 | Female | 3.11 | 5.56 | 3.71 | 4.43 | 5.83 | 4.39 |
| 20 | 19 | Male | 5.22 | 5.56 | 5.00 | 4.00 | 7.00 | 5.61 |
| 21 | 21 | Female | 5.44 | 4.78 | 2.86 | 5.29 | 5.17 | 4.44 |
| 22 | 21 | Female | 2.11 | 3.78 | 1.86 | 2.86 | 3.33 | 2.61 |
| 23 | 20 | Female | 3.50 | 5.22 | 2.71 | 3.00 | 5.83 | 3.59 |
| 24 | 19 | Female | 4.33 | 5.33 | 5.57 | 5.29 | 5.33 | 5.06 |
| 25 | 24 | Female | 4.78 | 6.22 | 4.29 | 3.57 | 6.67 | 4.61 |
| 26 | 20 | Female | 2.89 | 4.89 | 2.71 | 5.29 | 5.33 | 3.83 |
| 27 | 19 | Female | 3.56 | 4.56 | 3.57 | 4.29 | 5.17 | 3.94 |
| 28 | 19 | Male | 5.11 | 5.11 | 1.00 | 3.57 | 6.50 | 3.78 |
| 29 | 19 | Female | 3.67 | 4.00 | 2.29 | 4.43 | 5.00 | 3.50 |
| 30 | 18 | Female | 5.00 | 5.56 | 3.86 | 5.29 | 5.00 | 4.72 |
|  |  |  |  |  |  |  |  |  |
| **Mean** | 20.13 |  | 4.09 | 4.97 | 3.41 | 4.26 | 5.42 | 4.13 |
| **SD** | 2.18 |  | 1.13 | 0.70 | 1.44 | 0.98 | 0.77 | 0.91 |
| **Range** | [18, 27] |  | [2.00, 5.78] | [3.44, 6.44] | [1.00, 5.86] | [1.71, 5.57] | [3.33, 7.00] | [2.17, 5.67] |

**Appendix C**

For each recording, we used a numerical optimization technique to estimate the difference in key between the original song and the participant’s recording. With the assumption that the relative pitch contours are similar, key difference can be estimated as a constant bias between the two representations. Research assistants began by finding the section in the original song that corresponds with the participant’s recording. They then estimated the discrete pitches of both the original song fragment and the participant’s song fragment to the nearest semitone in the Twelve-Tone Equal Temperament Tuning System (12-TET). The pitch information was encoded in integer semitone units (e.g. A4 = 440 Hz was represented as $4\times12+9=57$). Since our objective was to estimate overall key difference, we identified the key transpose offset, $b$, in integer semitones, that minimized the error between the recording and the original song, averaged over the entire recording. Thus, if a participant’s key drifted during the recording, our calculation would be based on the average error across the entire fragment, rounded to the nearest semitone. In summary, we modeled the pitch error of each sung fragment as the Mean Absolute Error (MAE) between the original and transposed participant pitch vectors, rounded to the nearest semitone. Assuming $o,p\in Z^{n}$ are the pitch vectors, the bias was defined as:


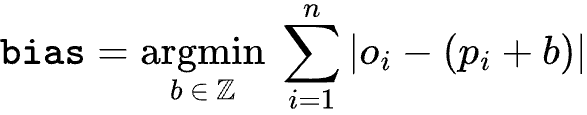


where *o_i_* is the *i*^th^ note of the original song fragment and *p_i_* is the *i*^th^ note of the produced song fragment. Because of the modular structure of keys in 12-TET tuning, we normalized this bias into the range [-5, 6] by removing the octave information. For example, if the minimized bias was +11, that means that the participant recording should be raised 11 semitones to best match the original recording. However, since we are ignoring octave information, transposing 11 semitones up is equivalent to transposing 1 semitone down. Thus, the key difference measure would be converted to -1. This is captured by the equation:


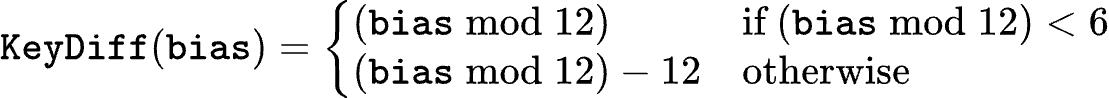

Supplement: Supplementary file 1 — Supplementary file1 (DOCX 35 KB) [file 13414_2024_2936_MOESM1_ESM.docx]
